# Free Sugars and Source-Resolved Sugar-Sweetened Beverage Consumption in Geographically Diverse Young Adults at an English University (2015–2016): Associations with Adiposity and Body Composition

**DOI:** 10.3390/nu18213552

**Published:** 2026-10-28

**Authors:** Antonio Peña-Fernández, Tomás Cámara-Pastor, M. Ángeles Peña Fernández

**Affiliations:** 1 Area of Legal and Forensic Medicine, Department of Surgery, Medical and Social Sciences, Faculty of Medicine and Health Sciences, University of Alcalá, Ctra. Madrid-Barcelona, Km. 33.6, 28871 Alcalá de Henares, Spain; 2 Leicester School of Allied Health Sciences, De Montfort University, Leicester LE1 9BH, UK; 3 Scientific Computation Research Institute (SCRIUR), University of La Rioja, 26006 Logroño, Spain; 4 Department of Biomedical Sciences, Faculty of Pharmacy, University of Alcalá, Crta. Madrid-Barcelona, Km. 33.6, 28871 Alcalá de Henares, Spain

**Keywords:** free sugars, sugar-sweetened beverages, body composition, adiposity, visceral fat, university students, young adults, United Kingdom

## Abstract

Background/Objectives: Free sugars and sugar-sweetened beverages (SSBs) remain important public-health targets, yet source-specific exposure and its relationship with body composition are poorly characterised in young adults attending English universities. Methods: This historical cross-sectional study examined 111 students aged 18–22 years at De Montfort University, Leicester, in 2015–2016. Dietary intake over approximately four weeks was assessed using an adapted EPIC-Norfolk food-frequency questionnaire processed in Nutritics. Free sugars were obtained from the native Nutritics variable, while strict SSB intake was reconstructed from 441 FoodName-level records, excluding diet/no-sugar beverages and 100% fruit/vegetable juice. Associations with BMI, body-fat percentage and BIA-derived visceral-fat index were estimated using HC3 robust multivariable models; adjusted inference included 109 participants. Results: Median free-sugar intake was 43.2 g/day (8.20% of total energy), 33.3% of participants had intakes ≥10% of total energy, and 81.1% consumed strict SSBs. Regular soft drinks/cola contributed 62.3% of total strict SSB intake. No statistically significant association was detected for either primary exposure with any adiposity outcome. For example, each 5-percentage-point higher free-sugar contribution was associated with a +0.50 percentage-point difference in body fat (95% CI −0.68 to 1.68), while a 1-SD higher log-transformed strict SSB intake was associated with a +0.87 percentage-point difference (95% CI −1.14 to 2.88). Conclusions: These confidence intervals were not sufficiently narrow to exclude potentially meaningful effects. The findings represent historical 2015–2016 estimates rather than current prevalence and should not be interpreted as evidence of no association. Larger prospective studies with validated source-specific exposure assessment and repeated body-composition measurements are warranted.

## 1. Introduction

Young adulthood represents a critical period in the life course for the establishment and consolidation of dietary behaviours. Between late adolescence and the early twenties, individuals commonly acquire greater autonomy over food purchasing, meal preparation, beverage choice and expenditure while simultaneously experiencing changes in residence, social networks, academic workload, sleep, physical activity and alcohol consumption [1,2,3]. Entry into higher education can therefore alter both the nutritional environment and the determinants of food choice. Importantly, behaviours established during this period may persist beyond university and contribute to longer-term cardiometabolic trajectories [1,2,3].

University students consequently represent a distinct population for nutritional surveillance rather than simply a younger subset of the adult population. Studies conducted in the United Kingdom have identified marked heterogeneity in student dietary behaviours, including differences in meal patterns, convenience-food intake, dietary quality and nutrient adequacy [4,5,6]. These behaviours occur within increasingly diverse university environments. Recent evidence from an English university population has highlighted substantial demographic heterogeneity in lifestyle and health-related characteristics [7]. Such diversity is epidemiologically relevant because dietary exposure may be shaped by multiple interacting influences, including previous food environments, family practices, migration history, residence, food availability, economic constraints and the transition to independent living.

Free sugars and sugar-sweetened beverages (SSBs) are among the dietary exposures of greatest public-health relevance in this context. Free sugars are conceptually distinct from total sugars. The Scientific Advisory Committee on Nutrition (SACN) defines free sugars as monosaccharides and disaccharides added to foods by manufacturers, cooks or consumers, together with sugars naturally present in honey, syrups and unsweetened fruit juices; lactose naturally present in milk and milk products and sugars contained within the intact cellular structure of foods are excluded [8]. The World Health Organisation (WHO) uses a closely aligned definition [9]. This distinction is important because total sugars combine nutritionally heterogeneous sources and should not be interpreted as equivalent to discretionary or freely available sugar exposure.

At the time the present cohort was studied, SACN recommended that average population free-sugar intake should not exceed 5% of dietary energy and that consumption of SSBs should be minimised [8]. WHO recommends reducing free sugars to <10% of total energy, with a conditional recommendation for further reduction to <5% [9]. National Diet and Nutrition Survey (NDNS) data corresponding closely to the study period showed that free-sugar exposure in the UK remained substantially above recommended levels. In analyses of the 2014/2015–2015/2016 NDNS, median free-sugar intake among adults aged 19–64 years was approximately 45 g/day and around 9–10% of energy, depending on the operational definition applied [10]. These national estimates, however, do not characterise the specific nutritional environment encountered during the transition into university.

The relationship between free sugars, SSBs and adiposity is supported by several complementary lines of evidence. Sugars consumed in liquid form may produce incomplete compensatory reductions in subsequent energy intake, facilitating positive energy balance when consumed regularly [8]. Meta-analyses of randomised trials and prospective cohorts have associated changes in dietary sugar intake with corresponding changes in body weight [11]. SSB consumption has additionally been associated with obesity, type 2 diabetes and cardiometabolic risk [12], and systematic reviews have generally supported positive associations between habitual SSB exposure and weight gain in both adults and children [13,14]. Conversely, replacing caloric SSBs with non-caloric alternatives has been associated with small favourable effects on body weight [15].

BMI, however, captures only one dimension of adiposity. Body-fat percentage and the regional distribution of adipose tissue may provide information that is not captured by body mass alone. This is particularly relevant to visceral adiposity, which is metabolically important and can vary substantially among individuals with similar BMI. SSB consumption has been associated with visceral adipose tissue in adult populations [16], while longitudinal data from the Framingham Heart Study demonstrated greater six-year visceral-fat accumulation among individuals with higher SSB consumption [17]. These findings provide a strong rationale for considering body-composition outcomes alongside conventional BMI.

Evidence specifically addressing free sugars and SSBs in university-age populations is considerably less developed. High SSB exposure has been documented among US college students [18], while added-sugar consumption and its relationship with BMI have been examined among South African undergraduates [19]. Within the UK, university studies have predominantly focused on broader eating behaviours, empirically derived dietary patterns, nutritional adequacy or general lifestyle characteristics [4,5,6,7]. A large study of undergraduate students in northern England, for example, examined energy and nutrient intake in relation to dietary reference values and general-population data, but did not undertake source-resolved SSB classification or examine free sugars in relation to measured body composition [6].

Accordingly, peer-reviewed evidence specifically characterising free sugars together with source-resolved SSB exposure and measured body composition in English university students remains sparse. To our knowledge, no previous study has combined a native free-sugar estimate, FoodName-level resolution of SSB exposure and measured BMI, body-fat percentage and visceral-fat status within the same geographically diverse English university cohort.

This methodological distinction is important. Broad dietary categories such as “soft/energy/sports drinks” can contain regular sugar-containing beverages alongside diet, zero-sugar and otherwise low-calorie products. Treating such categories as interchangeable with SSB exposure can therefore introduce exposure misclassification. Similarly, 100% fruit juice contributes free sugars under SACN and WHO definitions but is not conventionally classified as an SSB. Separating nutrient-level exposure from beverage-source exposure provides a more interpretable account of how free sugars enter the diet and which beverage sources contribute to overall exposure.

The present cohort offers several additional features that make this question particularly informative. Participants were restricted to a narrow age range of 18–22 years, thereby reducing age-related heterogeneity while focusing on a developmental period during which food-related autonomy is being established. The cohort was also geographically diverse, with substantial representation from African, Asian and European backgrounds. Geographical background was not regarded as a proxy for ethnicity, culture, religion, socioeconomic status or migration experience; rather, it was used as a broad demographic descriptor and adjustment variable. This composition therefore permits the dietary exposures of interest to be examined within a university cohort comprising participants from several geographical backgrounds, without implying representativeness of other university populations.

The novelty of the present study therefore lies not in any single analytical feature, but in their combination: a narrowly defined young-adult population; substantial geographical diversity; native Nutritics-derived free-sugar data; a complete FoodName-level audit of beverage exposure; explicit separation of strict SSBs, diet/no-sugar beverages and 100% fruit/vegetable juice; measurement of BMI and body composition; and a defined inferential framework incorporating robust standard errors, multiplicity control and extensive sensitivity analyses.

The primary objectives were consequently to: (i) quantify free-sugar intake in g/day and as a percentage of total energy; (ii) reconstruct a strict, source-resolved SSB exposure from the original food-level dietary records; and (iii) determine whether free-sugar and strict SSB exposure were associated with BMI, body-fat percentage and visceral-fat index after adjustment for age, sex, geographical background and total energy intake. A secondary objective was to investigate 100% fruit/vegetable juice independently of strict SSB exposure. The analysis was deliberately restricted to these sugar- and beverage-specific questions in order to distinguish it from broader dietary-pattern and nutrient-focused investigations of the same cohort.

## 2. Materials and Methods

### 2.1. Study Design, Setting and Participants

This study is a historical cross-sectional analysis of the university component of a wider biomonitoring and nutritional investigation undertaken at De Montfort University (DMU), Leicester, UK, during the 2015/2016 academic year, with participant data collection conducted in early 2016 [20]. For the present analysis, only quality-controlled dietary information and the anthropometric and body-composition variables required to address the free-sugar/SSB research question were considered.

Participants aged 18–22 years were recruited through multiple complementary routes across De Montfort University (DMU), Leicester, UK, during the 2015/2016 academic year. Recruitment activities extended across the university’s four faculties: Art, Design and Humanities; Business and Law; Health and Life Sciences; and Technology. Participation was open to eligible students irrespective of their faculty or academic programme. Study information was disseminated during scheduled teaching activities, through Blackboard virtual-learning environments, study posters and other recruitment materials, and with the assistance of student and course representatives [20]. The proximity of the four faculties within DMU’s Leicester city-centre campus facilitated access to the study’s assessment facilities. As recruitment channels overlapped, the total number of students reached could not be reliably established, and a formal response rate was not calculated.

Students who expressed interest received written information about the study and were offered flexible face-to-face assessment appointments. A £5 voucher was provided under an approved protocol amendment in recognition of the time involved in participation. Participation was voluntary, and written informed consent was obtained before undertaking any study procedure.

Assessments were conducted by appointment in the Biomedical and Environmental Health Research Laboratory, located in the Hawthorn Building, Leicester School of Allied Health Sciences, De Montfort University. Assistance with completion of study materials was available when required. Eligibility for the university component comprised enrolment at DMU, age 18–22 years, provision of written informed consent and general good health. Participants with chronic medical conditions requiring ongoing treatment, those receiving regular medical treatment, or those with a history of surgery were not eligible for the parent study. Inclusion in the present nutritional analysis additionally required dietary records of sufficient completeness and internal consistency to support quality-controlled processing. The final analytical cohort comprised 111 participants.

Formal ethical approval was granted by the Faculty Research Ethics Committee, Faculty of Health and Life Sciences, De Montfort University (Ref. 1674) on 11 January 2016. Recruitment, informed consent and the study procedures contributing data to the present analysis were undertaken following this approval. The study was conducted in accordance with the Declaration of Helsinki. Subsequent protocol amendments were approved through the institutional ethics process. Data collection was concentrated within a single continuous period from January to mid-March 2016 and was completed before the Easter vacation. This relatively short collection window reduced temporal and seasonal heterogeneity in dietary assessment.

Geographical background was derived from the recorded geographical region of birth and categorised as Africa, Asia, Europe or Other. The Other category comprised two participants, one recorded as Caribbean and one as Central America. Geographical background was used exclusively as a broad demographic descriptor and adjustment variable and was not interpreted as a proxy for ethnicity, genetic ancestry, culture, religion, socioeconomic circumstances or migration history. Because only two participants were classified as Other, this category was retained in descriptive analyses but excluded from primary adjusted inference to avoid unstable parameter estimation, yielding an inferential sample of 109 participants.

The retained analytical dataset comprised 111 participants with quality-controlled dietary and body-composition data, all of whom were included in the descriptive analyses. The primary adjusted models included 109 participants, following exclusion of two individuals classified in the “Other” geographical-background category owing to its limited sample size. No additional exclusions due to missing analytical variables were required for these models. The available historical records did not permit reliable reconstruction of the numbers of students initially approached, screened, assessed or excluded before formation of the retained analytical dataset.

### 2.2. Anthropometric and Body-Composition Assessment

Age and sex were recorded for each participant. Standing height was measured during the study appointment by a qualified nurse using a seca 213 portable stadiometer (seca GmbH & Co. KG, Hamburg, Germany; 1 mm graduation). Height was subsequently entered into the Tanita SC-330-S body-composition analyser. Body weight and body-composition variables were obtained using the Tanita SC-330-S (Tanita Corporation, Tokyo, Japan), which estimates body composition by bioelectrical impedance analysis (BIA) [21,22]. Manufacturer specifications report a 0.1 kg weight graduation, ±0.2 kg weight accuracy at first calibration and ±2% impedance accuracy [22].

In addition to the general health eligibility criteria described above, BIA was not undertaken in individuals with pacemakers or other implanted electronic medical devices, in accordance with the manufacturer’s safety instructions. Participants received standardised pre-assessment instructions intended to reduce short-term variability in BIA measurements. They were asked to abstain from food, alcoholic beverages, coffee and other caffeinated beverages, smoking and physical exercise for at least 8 h before assessment; water was permitted. Measurements were obtained with participants barefoot, following the manufacturer’s operating procedure. These conditions were intended to reduce variation attributable to recent food and fluid intake and exercise [21].

The analyser provided estimates of body-fat percentage, total body water, muscle mass, bone mass, visceral-fat rating/index and basal metabolic rate. The three primary adiposity outcomes were BMI, body fat (%) and visceral-fat index. BMI was independently recalculated from measured height and weight according to:

BMI (kg/m^2^) = weight (kg)/height^2^ (m^2^).

The SC-330 expresses visceral-fat rating on a 1–59 scale, with the manufacturer identifying values 1–12 as within its reference range and values ≥13 as elevated [22]. The output is a BIA-derived device estimate rather than a direct anatomical measurement of visceral adipose tissue. In the present cohort, values ranged only from 1 to 9; consequently, dichotomisation at the manufacturer’s elevated threshold would have produced no elevated group. Visceral-fat index was therefore retained as a continuous outcome, with analyses involving this restricted-range measure interpreted as exploratory. Its distribution is additionally reported in Supplementary Figure S1.

### 2.3. Dietary Assessment and Data Reconstruction

Dietary intake over approximately the preceding four weeks was assessed using an expanded semi-quantitative food-frequency questionnaire (FFQ) developed for the parent study. Its core food-frequency structure was based on an adapted version of the EPIC-Norfolk FFQ, version 6 (CAMB/PQ/6/1205), whose underlying nutritional methodology has been described previously [23]. Questionnaire development was also informed by a semi-quantitative FFQ previously used by the research group in young adults, which incorporated small, medium and large portion-size categories and had been evaluated against repeated 24 h dietary recalls. That validation applied to the earlier questionnaire that informed instrument development; the expanded FFQ administered in the present cohort was not independently revalidated against a reference dietary-assessment method.

The administered questionnaire contained more than 130 food and beverage items. The original four-week reference period and the core frequency- and portion-response framework were retained. Participants reported consumption using predefined frequency categories ranging from no or infrequent monthly intake to multiple occasions per day and selected small, medium or large portions relative to food-specific medium serving sizes. Adaptation of the questionnaire consisted principally of adding food and beverage items to improve dietary coverage for the geographically heterogeneous university population. These additions were intended to capture foods consumed within the study setting and were not used to assign foods or dietary behaviours according to participants’ geographical background. Food-composition interpretation was informed by established UK resources, including McCance and Widdowson’s *The Composition of Foods* [24].

Completed FFQs were reviewed for sufficient completeness and internal interpretability before inclusion in the nutritional analysis and were processed using Nutritics^®^ Research Edition, version 5.7 (Nutritics Ltd., Dublin, Ireland). Nutritics processing converted the reported frequency and portion selections into food-level weekly intake estimates expressed in grams. In the retained source export, estimated food intake was stored as grams per week. Daily intake was therefore derived once as:

g/day = g/week/7.

Raw and derived fields were maintained separately throughout processing to preserve provenance and analytical traceability. The retained food-level dataset contained participant identifier, FoodName, original food category and estimated gram intake, from which the free-sugar and source-resolved beverage exposures used in the present analysis were reconstructed.

### 2.4. Definition and Derivation of Free Sugars

Total sugars and free sugars were treated as conceptually distinct dietary variables. Free sugars were not reconstructed by summing individual sugar components, such as glucose, fructose or sucrose. The original Nutritics batch export contained a native Free Sugars(g) field alongside Sugars(g) and individual sugar variables.

Nutritics defines free sugars as sugars added to food by a manufacturer, cook or consumer together with sugars naturally present in fruit juice, honey and syrups, while excluding sugars naturally present in milk and milk products and those contained in fruit and vegetables [25]. Importantly, Nutritics also states that the database breakdown of total sugars into individual components such as glucose and fructose is incomplete and should be regarded as indicative [25]. The original Nutritics-derived Free Sugars(g) values were retained without recalculation. The subsequent *FoodName*-level review was used to classify beverage sources, as detailed in Section 2.5 and Supplementary Table S1, rather than to modify the original free-sugar estimates.

The weekly Nutritics Free Sugars(g) variable was divided by seven to derive free-sugar intake in g/day. Free sugars as a percentage of total energy were calculated as:

Free sugars (% total energy) = 100 × [(free sugars, g/day × 4 kcal/g)/total energy intake, kcal/day].

Percentage total energy was selected as the primary free-sugar exposure because it characterises sugar intake relative to overall dietary energy consumption and reduces the extent to which exposure simply represents greater total food intake.

For descriptive purposes, free-sugar percentage was additionally categorised as <5%, 5–<10% and ≥10% of total energy. These were used as reference bands only and were not interpreted as individual diagnostic or compliance categories.

Free-sugar percentages were calculated using total reported energy intake, as participant-level energy excluding ethanol was not available. Accordingly, the <5% category was used as a descriptive reference band and was not interpreted as an exact assessment of adherence to the SACN population recommendation, which is expressed relative to energy excluding ethanol [26].

### 2.5. Source-Resolved Classification of Sugar-Sweetened Beverages

A central methodological objective was to avoid equating broad beverage categories with true SSB exposure. Strict SSB intake was therefore reconstructed independently from the original FoodName-level dietary records.

A total of 441 unique FoodNames were reviewed using an auditable classification procedure. The original FoodName, source category and classification decision were retained for every record. The complete FoodName-level classification is provided in Supplementary Table S1.

The primary strict SSB definition included regular sugar-containing carbonated soft drinks and cola; regular sugar-containing lemonade and comparable soft drinks; sugar-containing energy drinks; sugar-containing sports drinks; sugar-sweetened fruit or juice drinks that were not 100% juice; and sugar-containing squash or cordials.

The definition excluded diet, light, zero, sugar-free, no-sugar and equivalent variants; plain or carbonated water; 100% fruit/vegetable juice; genuine smoothies; milkshakes and dairy-based beverages; meal-replacement drinks; alcoholic beverages; and tea or coffee unless explicitly identified as a prepared sugar-sweetened beverage.

Classification was deliberately conservative. Three beverage FoodNames could not be assigned unequivocally from the historical information available and were therefore excluded from the primary strict SSB definition rather than classified through assumptions about product formulation. Contemporary commercial formulations were not used to retrospectively infer the composition of products consumed during 2015–2016. The three ambiguous items were subsequently incorporated into a dedicated robustness analysis.

Commercial product names, where present, are retained in the Supplementary FoodName audit solely to document the classification process and support reproducibility; their inclusion does not imply endorsement by the authors.

Strict SSB intake was aggregated by participant from the original food-level estimates expressed in grams per week and divided by seven to obtain g/day. These values represent estimated beverage intake by mass rather than grams of sugar. No conversion from grams to millilitres or additional dilution assumptions for squash/cordials were applied during the source-level reconstruction. Accordingly, strict SSB intake was treated as a beverage-mass exposure rather than a direct estimate of beverage-derived free sugars. Five beverage-source categories were retained for descriptive interpretation: regular soft drinks/cola, squash/cordials, sports drinks, energy drinks and sugar-sweetened fruit drinks.

### 2.6. 100% Fruit/Vegetable Juice

One hundred percent fruit/vegetable juice was deliberately separated from strict SSB intake. This distinction reflects the fact that sugars naturally present in juice satisfy the definition of free sugars [8,9], whereas unsweetened 100% juice is not conventionally equivalent to a beverage sweetened with added sugars.

The historical FoodName data were audited to distinguish 100% juice from sugar-sweetened fruit drinks and juice drinks. No genuine smoothie FoodNames were identified in the source dataset. The publication-facing variable is therefore described as 100% fruit/vegetable juice intake, rather than fruit juice/smoothie intake.

Juice was analysed as a secondary exposure and did not contribute to the strict SSB variable.

### 2.7. Exposures, Outcomes and Covariates

Before implementation of the definitive statistical analyses, the research team identified two primary dietary exposures for the present retrospective investigation: (a) free sugars as a percentage of total energy, parameterised per 5-percentage-point increase; and (b) strict SSB intake in g/day.

Strict SSB intake contained structural zeroes and demonstrated marked positive skewness. It was therefore transformed using:

log [1 + SSB intake (g/day)]

and subsequently standardised. Primary SSB coefficients thus represent the outcome difference associated with a 1-SD increase in log1p-transformed strict SSB intake.

Three primary adiposity outcomes were selected as part of the initial analytical framework: BMI (kg/m^2^), body fat (%) and visceral-fat index.

The primary adjustment set comprised age, sex, geographical background and total energy intake. Total energy was log-transformed. For free sugars expressed as a percentage of total energy, energy adjustment allowed associations with adiposity to be estimated at comparable levels of reported total energy intake, thereby focusing on dietary energy composition [27]. For strict SSB intake, the adjusted models estimated associations conditional on total energy intake. As total energy may also contribute to the relationship between SSB consumption and adiposity, complementary models without energy adjustment were examined as sensitivity analyses.

Primary adjusted inference was restricted to participants classified as Africa, Asia or Europe. The two participants in the Other category remained in descriptive summaries but were excluded from the primary models, yielding an inferential sample of 109.

### 2.8. Statistical Analysis

The present study represents a retrospective, sugar-focused secondary analysis of data collected in early 2016 as part of a broader biomonitoring and nutritional investigation. The analytical framework, including the primary dietary exposures, adiposity outcomes, adjustment covariates, and model specifications, was established before the statistical analyses were conducted.

All definitive analyses were performed using R version 4.4.2 (R Foundation for Statistical Computing, Vienna, Austria) [28]. The analytical workflow and corresponding outputs were finalised before manuscript preparation, and all numerical results reported in this study were derived from these definitive outputs.

Continuous variables were summarised using mean ± standard deviation and median with interquartile range where appropriate. Categorical variables are presented as *n* (%). Reporting both arithmetic and distribution-resistant summaries was particularly important for dietary variables because several beverage exposures displayed strong right-skewness.

Associations between the two primary exposures and BMI, body fat (%) and visceral-fat index were estimated using multivariable linear regression. Heteroskedasticity-consistent HC3 standard errors were used for primary inference because of their favourable finite-sample properties and robustness to heteroskedastic residual variance [29]. Each model included age, sex, geographical background and log-transformed total energy intake. Sex and geographical background were entered as categorical covariates, with female participants and Europe, respectively, serving as the reference categories.

Both unstandardised regression coefficients (*B*) and standardised coefficients (*β*), with 95% confidence intervals, were retained. Standardised coefficients were calculated as *B* × SD(exposure)/SD(outcome), using the exposure scale specified in each model. They express the adjusted outcome difference in standard-deviation units per 1-SD difference in exposure. Unstandardised coefficients were reported in the original outcome units to facilitate clinical interpretation.

The primary inferential family comprised the two dietary exposures and three adiposity outcomes defined in the analytical framework before implementation of the definitive statistical models, yielding six statistical tests. Benjamini–Hochberg false-discovery-rate correction was applied jointly across these six tests [30]. Both raw *p*-values and BH-FDR-adjusted *q*-values are reported. Interpretation was based on effect estimates, confidence intervals and the defined multiplicity framework rather than isolated nominal *p*-values.

A separate secondary three-test family assessed 100% fruit/vegetable juice in relation to the same three outcomes, with BH-FDR correction across those tests.

Sensitivity analyses evaluated free sugars per 25 g/day, with and without adjustment for total energy; free sugars as a percentage of total energy without energy adjustment; strict SSB intake per 100 g/day on its original scale; SSB consumer versus non-consumer status; strict SSB models without total energy adjustment; inclusion of the two participants in the Other geographical-background category as a fourth category (*N* = 111); quadratic exposure terms; influence-deleted refitting based on Cook’s distance; and an expanded SSB definition incorporating the three ambiguous FoodNames.

Because visceral-fat index is an ordered integer variable, proportional-odds ordinal regression was additionally undertaken as a sensitivity analysis. The models converged; however, the available formal diagnostic did not yield a valid confirmation of the proportional-odds assumption. Ordinal models were therefore retained as supportive sensitivity analyses only, while the HC3 linear models remained the primary analytical approach.

Model diagnostics included assessment of residual distribution, heteroskedasticity, specification, variance-inflation factors, leverage, Cook’s distance and studentized residuals.

Dietary data quality control included verification of weekly-to-daily intake conversions, internal consistency checks of energy and sugar variables, and examination of extreme observations against the source records and model-influence diagnostics. Participant-specific physical-activity information was insufficiently complete for Goldberg classification [31,32]; therefore, no participant was excluded solely on the basis of reported energy intake.

The study is reported in accordance with the principles of the STROBE statement for observational studies [33].

## 3. Results

### 3.1. Participant Flow, Characteristics and Free-Sugar Exposure

The retained analytical cohort comprised 111 students aged 18–22 years, with a mean age of 20.4 ± 1.2 years, all of whom were included in descriptive analyses. The primary adjusted models included 109 participants (98.2%), following exclusion of two participants (1.8%) from the “Other” geographical-background category. No further exclusions due to missing analytical variables were required. Seventy-eight participants were female (70.3%) and 33 were male (29.7%). Geographical background was Africa for 41 participants (36.9%), Asia for 41 (36.9%), Europe for 27 (24.3%) and Other for two (1.8%), demonstrating substantial geographical diversity within a narrowly defined young-adult cohort (Table 1).

Mean total energy intake was 2524.1 ± 1256.1 kcal/day, with a median of 2245.4 kcal/day. Mean total-sugar intake was 144.0 ± 91.7 g/day and median intake was 121.7 g/day. In comparison, mean free-sugar intake was 64.2 ± 59.5 g/day and median intake was 43.2 g/day, illustrating the substantial quantitative distinction between total and free sugars.

**Figure 1:**
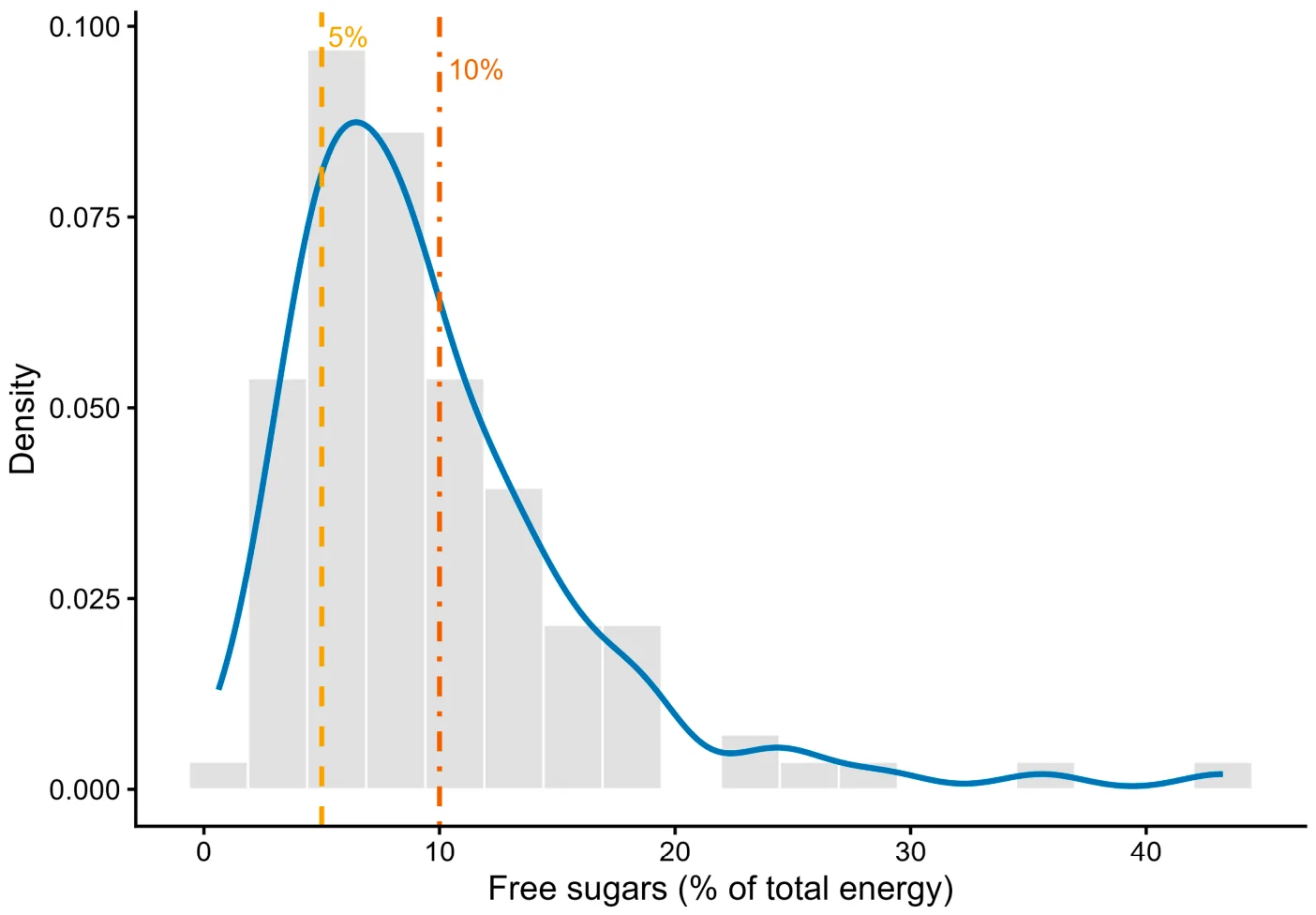
The distribution of free sugars as a percentage of total energy intake in 111 young adults attending an English university. The vertical reference lines at 5% and 10% of total energy are shown for descriptive context and correspond to WHO free-sugar reference thresholds [9]. The 5% line should not be interpreted as an assessment of individual compliance with current UK SACN guidance [26], which expresses the population dietary reference value relative to energy excluding ethanol.

Mean free-sugar intake represented 9.81 ± 6.65% of total energy, with a median of 8.20% [IQR 5.38–12.28%]. The distribution was strongly right-skewed and ranged from 0.62% to 43.25% of total energy (Figure 1; Supplementary Table S2).

Using the defined descriptive bands, 21 participants (18.9%) had a free-sugar intake < 5% of total energy, 53 (47.7%) had an intake between 5% and <10%, and 37 (33.3%) had an intake ≥ 10% (Supplementary Table S2). Thus, more than four in five participants had free-sugar exposure at or above 5% of total energy, while one in three had an intake ≥ 10%. These categories are descriptive rather than formal current-SACN compliance categories because energy excluding ethanol could not be reconstructed.

Mean BMI was 23.69 ± 4.09 kg/m^2^, mean body fat was 25.7 ± 10.4%, and mean visceral-fat index was 2.67 ± 2.02 (median 2; IQR 1–4). Visceral-fat index values ranged from 1 to 9, with 68 participants (61.3%) presenting values of 1 or 2 (Supplementary Figure S1).

Additional descriptive summaries by sex and geographical background (Africa, Asia and Europe) are provided in Supplementary Table S3. No subgroup-specific inferential models were fitted.

### 3.2. Source-Resolved Sugar-Sweetened Beverage Consumption

Twenty-one participants (18.9%) had zero strict SSB intake, while 90 (81.1%) consumed at least one beverage classified as a strict SSB. Mean strict SSB intake was 343.2 ± 452.9 g/day, whereas median intake was 159.1 g/day [IQR 45.4–483.9], demonstrating pronounced right-skewness (Table 2; Supplementary Figure S2).

**Figure 2:**
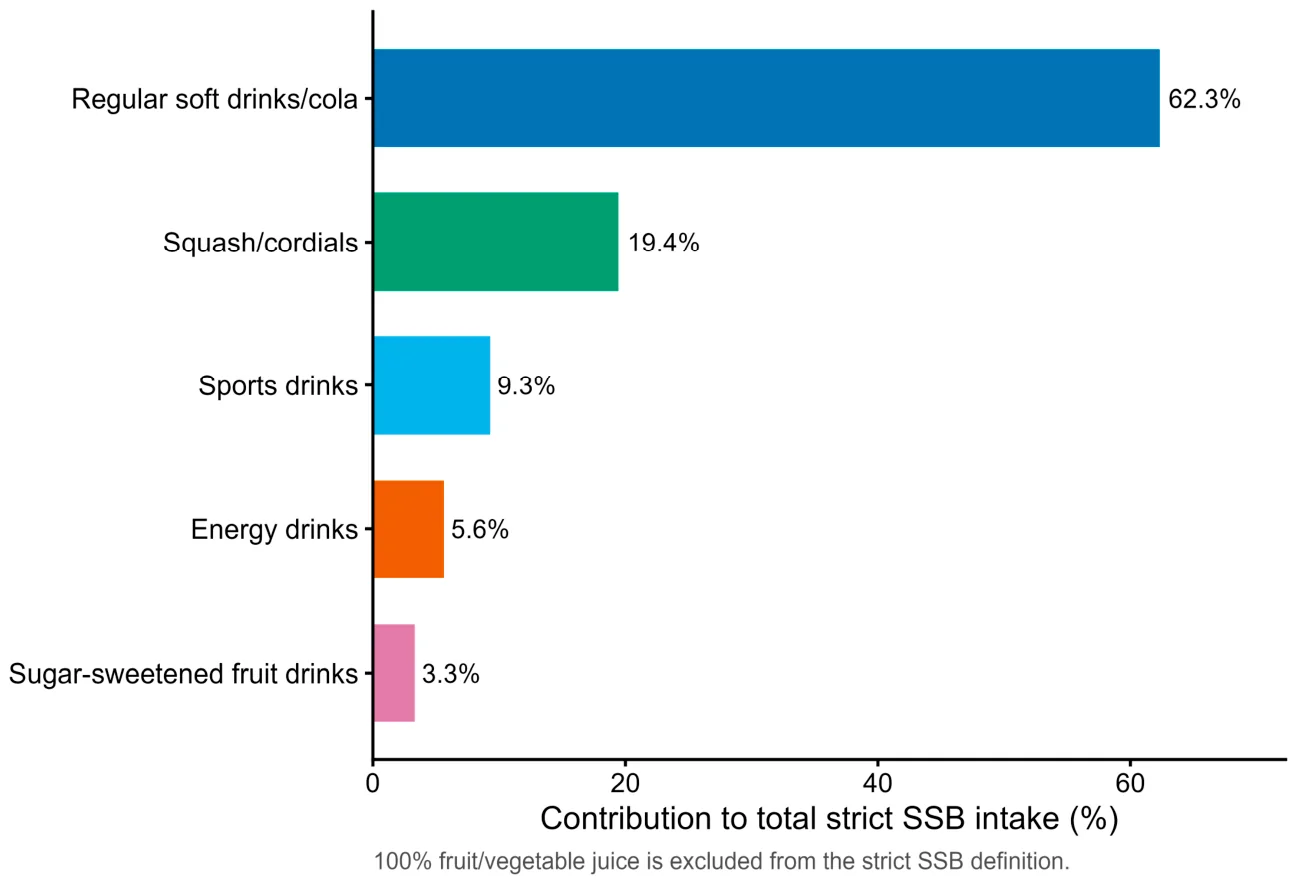
Contribution of source-resolved beverage subtypes to total strict sugar-sweetened beverage (SSB) intake. Regular soft drinks/cola contributed 62.3%, squash/cordials 19.4%, sports drinks 9.3%, energy drinks 5.6%, and sugar-sweetened fruit drinks 3.3%. One hundred percent fruit/vegetable juice was analysed separately and was not included in strict SSB intake.

Regular soft drinks and cola represented the dominant beverage source. They were consumed by 80 participants (72.1%) and accounted for 62.3% of total strict SSB intake by mass across the cohort. Squash/cordials contributed 19.4%, sports drinks 9.3%, energy drinks 5.6%, and sugar-sweetened fruit drinks 3.3% (Table 2; Figure 2).

Together, beverage sources other than regular soft drinks/cola accounted for 37.7% of estimated strict SSB intake in this cohort.

One hundred percent fruit/vegetable juice was consumed by 93 participants (83.8%). Mean intake was 43.2 ± 72.5 g/day and median intake was 22.9 g/day [IQR 1.4–46.4]. This exposure was maintained separately from strict SSB intake despite contributing to free-sugar exposure.

The complete *FoodName*-level classification process, comprising all 441 unique *FoodNames* and the three ambiguous beverage records, is provided in Supplementary Table S1.

### 3.3. Primary Associations with Adiposity and Body Composition

**Figure 3:**
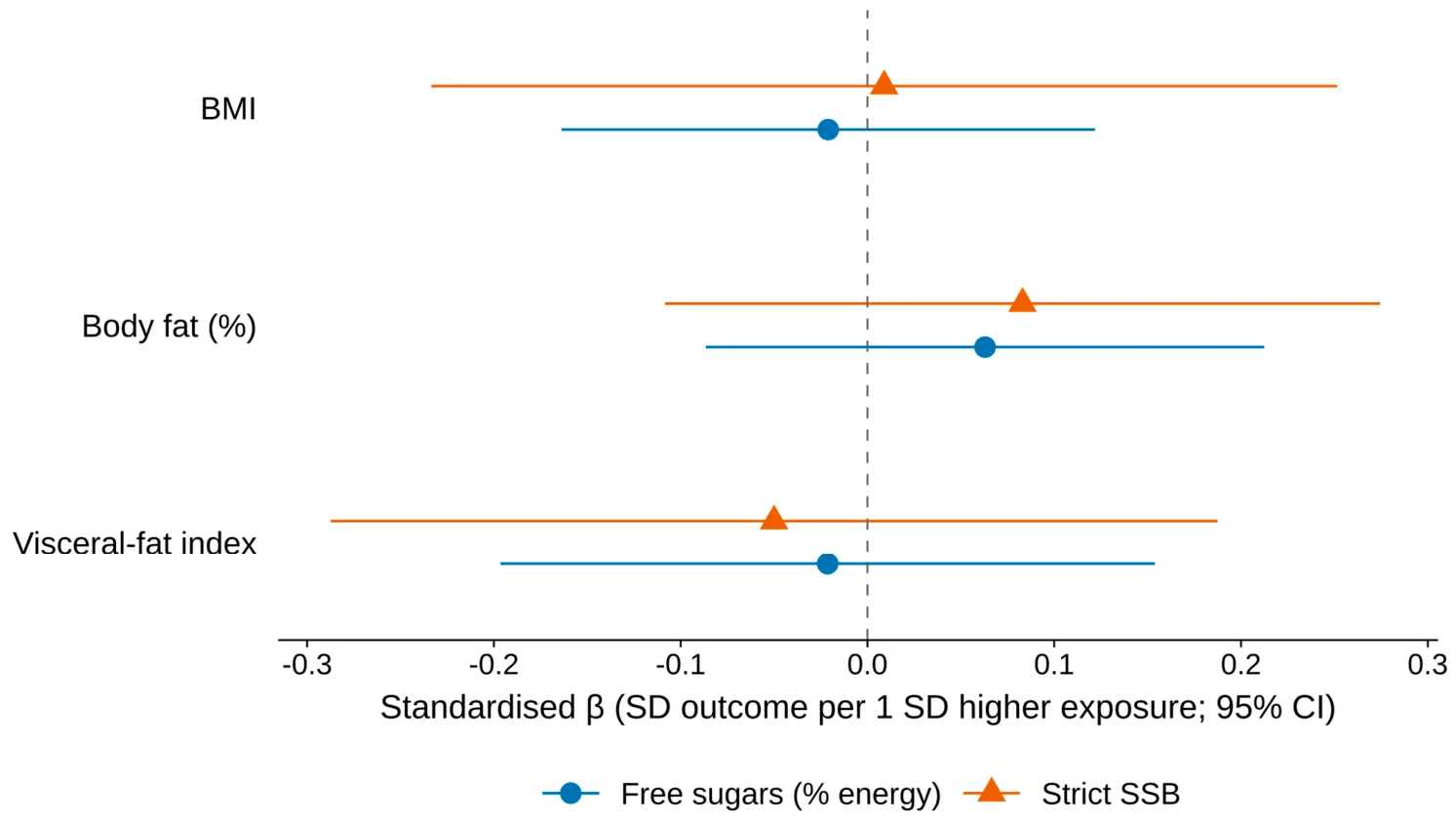
The standardised associations of free sugars and strict sugar-sweetened beverage (SSB) intake with adiposity and body composition (*n* = 109). The points show the standardised regression coefficients (*β*), calculated as *B* × SD(exposure)/SD(outcome), and the horizontal lines represent 95% confidence intervals. The models were adjusted for age, sex, geographical background and log-transformed total energy intake. The corresponding unstandardised coefficients, expressed in the original outcome units and exposure scales, are reported in Table 3.

None of the six prespecified primary exposure–outcome associations reached statistical significance after multivariable adjustment (Table 3; Figure 3).

For free sugars, each five-percentage-point higher contribution to total energy was associated with a BMI difference of −0.065 kg/m^2^ (95% CI −0.509 to 0.379; standardised β = −0.021; *p* = 0.771), a body-fat difference of +0.498 percentage points (95% CI −0.684 to 1.681; standardised β = 0.063; *p* = 0.405), and a visceral-fat-index difference of −0.032 (95% CI −0.297 to 0.232; standardised β = −0.021; *p* = 0.810).

For strict SSB intake, a 1-SD higher log1p-transformed exposure was associated with a BMI difference of +0.037 kg/m^2^ (95% CI −0.963 to 1.037; standardised β = 0.009; *p* = 0.942), a body-fat difference of +0.872 percentage points (95% CI −1.138 to 2.881; standardised β = 0.083; *p* = 0.392), and a visceral-fat-index difference of −0.100 (95% CI −0.576 to 0.376; standardised β = −0.050; *p* = 0.677).

Following joint BH-FDR correction across the six primary tests, all adjusted *q*-values were 0.942. Although the point estimates were generally small, the 95% confidence intervals encompassed associations in either direction, particularly for body-fat percentage.

### 3.4. Secondary and Sensitivity Analyses

Secondary analyses of 100% fruit/vegetable juice similarly provided no evidence of association with adiposity (Supplementary Table S5). Per 1-SD increase in log1p-transformed juice intake, the adjusted coefficients were +0.297 kg/m^2^ for BMI (95% CI −0.506 to 1.100; *p* = 0.464; *q* = 0.464), +0.868 percentage points for body fat (95% CI −0.773 to 2.510; *p* = 0.296; *q* = 0.445), and +0.232 for visceral-fat index (95% CI −0.199 to 0.663; *p* = 0.287; *q* = 0.445).

Alternative exposure parameterisations and energy-adjustment sensitivity analyses yielded no statistically significant associations (Supplementary Tables S6 and S7). For strict SSB intake, models without total energy adjustment yielded an estimated BMI difference of +0.025 kg/m^2^ (95% CI −1.042 to 1.092), a body-fat difference of +1.161 percentage points (95% CI −0.878 to 3.200), and a visceral-fat-index difference of −0.092 (95% CI −0.598 to 0.413) per 1-SD increase in log1p-transformed intake. These estimates were similar in direction and magnitude to those from the primary energy-adjusted models. For free sugars expressed per 25 g/day, the estimated body-fat difference was +0.637 percentage points (95% CI −0.373 to 1.646) with energy adjustment and +0.688 (95% CI −0.145 to 1.521) without energy adjustment. When free sugars were expressed per five percentage points of total energy, the corresponding body-fat estimate was +0.606 percentage points (95% CI −0.652 to 1.864) without energy adjustment, compared with +0.498 (95% CI −0.684 to 1.681) in the primary model. Complete comparisons across all three outcomes are provided in Supplementary Table S7. Alternative SSB parameterisations, consumer versus non-consumer classification and inclusion of the Other geographical-background category (*N* = 111) did not materially change the overall findings (Supplementary Table S6).

The conservative robustness analysis in which all three ambiguous beverage FoodNames were incorporated into an expanded SSB definition similarly produced no evidence of association (Supplementary Table S8). Under this expanded exposure, SSB associations yielded *p* = 0.802 for BMI, *p* = 0.216 for body fat and *p* = 0.893 for visceral-fat index. No test survived BH-FDR correction.

Model diagnostics are reported in Supplementary Table S9. Some evidence of non-normality and heteroskedasticity was present, supporting the prespecified use of HC3 robust standard errors. Multicollinearity was low, with maximum variance-inflation factors below approximately 1.8. Quadratic terms did not provide evidence of meaningful non-linearity, and influence-deleted refits did not materially alter the conclusions. The consistency of effect estimates across alternative exposure definitions and model specifications is summarised visually in Supplementary Figure S3.

Ordinal-regression sensitivity models for visceral-fat index also yielded no evidence of association. The odds ratio for a higher visceral-fat category was 1.045 (95% CI 0.813–1.342; *p* = 0.731) per five-percentage-point increase in free-sugar energy and 1.122 (95% CI 0.760–1.658; *p* = 0.562) per 1-SD increase in log-transformed strict SSB intake. Because the proportional-odds assumption could not be formally confirmed, these models were treated as supportive rather than primary (Supplementary Table S9).

## 4. Discussion

### 4.1. Principal Findings, Novelty and Methodological Contribution

The present study provides a detailed, source-resolved characterisation of free-sugar and sugar-sweetened beverage (SSB) exposure among geographically diverse young adults attending an English university in early 2016 and evaluates its associations with three complementary measures of adiposity. Four findings are particularly important. First, free-sugar exposure was substantial: median intake was 43.2 g/day and represented 8.20% of total energy, while one-third of participants had intakes ≥ 10% of total energy. Second, strict SSB consumption was widespread, with 81.1% of participants classified as consumers. Third, although regular soft drinks/cola represented the dominant source of strict SSB intake, 37.7% of estimated strict SSB intake by mass originated from other beverage categories. Fourth, none of the six primary adjusted models reached statistical significance. However, the 95% confidence intervals remained compatible with associations in either direction. Sensitivity and robustness analyses yielded broadly consistent findings.

The principal novelty of the study lies in the combination of population, exposure resolution and phenotype assessment rather than in any one of these components in isolation. The cohort was restricted to young adults aged 18–22 years, capturing a narrow developmental period during which individuals are transitioning from late adolescence into greater dietary autonomy. It was also geographically heterogeneous, with substantial representation from African, Asian and European backgrounds. Within this population, free sugars were quantified from the native Nutritics variable, beverage exposure was reconstructed from the original FoodName-level records, and adiposity was characterised using BMI together with body-fat percentage and visceral-fat index. To our knowledge, this combination has not previously been reported in a similarly geographically diverse English university cohort.

A particularly important methodological contribution is that the analysis moves beyond treating “sugar intake” as a single, homogeneous exposure. Free sugars and SSBs are related but non-equivalent constructs. Free sugars integrate sugars from multiple food and beverage sources, whereas strict SSB intake captures one behaviourally identifiable component of that exposure. Likewise, 100% fruit/vegetable juice contributes free sugars under UK and WHO definitions but differs compositionally and behaviourally from beverages to which sugars have been added. Preserving these distinctions enables questions to be addressed simultaneously at the nutrient and food-source levels.

This distinction is highly relevant from both food-science and public-health perspectives. Dietary guidance, reformulation, taxation, procurement and substitution strategies operate primarily through foods and beverages rather than through abstract nutrient totals. Two individuals may therefore have comparable free-sugar intake while deriving that exposure from substantially different food matrices and beverage behaviours. A nutrient-only analysis cannot identify those differences, whereas an aggregated beverage category can create a different problem by combining products with opposing sugar profiles.

For that reason, strict SSB intake was reconstructed directly from the source dietary records. All 441 unique FoodNames were audited, diet/no-sugar products were separated from sugar-containing alternatives, 100% fruit/vegetable juice was maintained outside the strict SSB definition, and no genuine smoothie FoodNames were identified. Importantly, three historically ambiguous beverage records were not assigned to the primary SSB definition on the basis of assumptions about contemporary commercial formulations. They were instead incorporated in a dedicated robustness analysis. The sensitivity analysis incorporating these three records yielded findings consistent with those obtained using the primary strict SSB classification.

The use of the native Nutritics free-sugar variable represents a second important strength of exposure assessment. Nutritics explicitly distinguishes free sugars from total sugars and states that the breakdown of total sugars into individual components such as glucose and fructose can be incomplete [25]. Retrospective reconstruction of free sugars from glucose, fructose, sucrose or related fields could therefore have introduced avoidable measurement error. The present analysis instead retained and independently verified the native Free Sugars(g) field from the original export.

The numerical distinction between total and free sugars in this cohort further demonstrates why this methodological choice matters. Mean total-sugar intake was 144.0 g/day, more than twice the mean free-sugar intake of 64.2 g/day. Total sugars incorporate sugars arising within markedly different food matrices and cannot be assumed to carry the same dietary meaning as free sugars. Analyses limited to total sugar could consequently obscure the exposure addressed by current UK and international public-health guidance.

### 4.2. Free-Sugar and Beverage Exposure in the UK and International Context

The magnitude of free-sugar exposure observed in this cohort should first be considered in relation to the UK nutritional environment in which the data were collected. Median free-sugar intake was 43.2 g/day, close to the approximately 45 g/day reported among UK adults aged 19–64 years in analyses of NDNS data covering 2014/2015–2015/2016 [10]. Similarly, median free sugars accounted for 8.20% of total energy in the present cohort, compared with approximately 9–10% in contemporary adult surveillance, depending on the operational definition used [10].

The free-sugar intake observed in this cohort was of a similar order of magnitude to that reported in contemporaneous UK adult surveillance, suggesting that the dietary exposure identified among these university students was also relevant within the wider nutritional context of the study period. Differences in study populations and dietary-assessment methods should be considered when interpreting these descriptive comparisons.

This interpretation is consistent with other English university research. Martin et al. examined 639 undergraduate students aged 18–24 years in northern England and reported that several aspects of their energy and nutrient intake were broadly comparable with general-population data [6]. That investigation was not designed to quantify native free sugars, reconstruct SSB exposure at FoodName level or examine body composition in the way undertaken here; nevertheless, it similarly challenges the assumption that all components of university-student diets are necessarily worse than population norms.

At the same time, similarity with general UK exposure should not be interpreted as reassuring. The public-health concern is precisely that excessive free-sugar intake has been widespread at the population level. In the present cohort, only 18.9% had free-sugar intake < 5% of total energy, while one-third had intake ≥ 10%. Nevertheless, the distribution illustrates substantial exposure heterogeneity and shows that relatively high free-sugar consumption was common rather than confined to a small number of extreme consumers.

More recent national surveillance indicates that the problem persists. In the 2019–2023 NDNS, mean free-sugar intake remained approximately 10.4% of energy among adults aged 19–64 years, while only 17% of this age group met the UK recommendation of no more than 5%. Among adolescents aged 11–18 years, mean free-sugar intake was approximately 10.9% of energy in boys and 12.0% in girls [34]. Because contemporary UK estimates use energy excluding ethanol whereas the present analysis uses total energy, these percentages should not be compared as if analytically identical. These more recent surveillance findings indicate that free-sugar exposure remains a relevant public-health concern in the UK. However, the present findings characterise dietary intake in a specific university cohort assessed in early 2016 and should not be interpreted as reflecting current consumption patterns among university students.

SSB consumption was similarly common in the present cohort. Eighty-one percent of participants consumed at least one strict SSB, although the marked right-skewness of intake produced a substantial difference between the mean of 343.2 g/day and median of 159.1 g/day. This distribution reinforces the value of reporting both consumer prevalence and quantitative exposure rather than reducing SSB behaviour to a binary variable.

International university evidence supports the broader conclusion that SSB consumption is common during this period of life, although direct numerical ranking between studies is rarely justified. Among 265 US college students investigated by West et al., 95% reported SSB consumption during the preceding month and 65% reported daily consumption [18]. Our estimate of 81.1% strict SSB consumers derives from a different dietary method, exposure window and classification system; therefore, it cannot validly be interpreted as evidence that US students consumed “more” or “less” than the present English cohort. The important commonality is that sugar-containing beverages represent a frequent exposure in university-age populations.

Nakhooda and Wiles similarly studied 387 South African undergraduates aged 18–25 years and reported associations between BMI and the frequency of consumption of selected foods containing added sugars [19]. Taste and price were also identified as important determinants of SSB purchasing and consumption. Their exposure assessment, population and statistical framework differed materially from ours: they evaluated selected sugar-containing foods and frequency measures rather than a source-resolved quantitative SSB exposure incorporated into energy-adjusted body-composition models. Apparently differing findings between the two studies should therefore not be interpreted as direct contradiction.

The source composition observed here is particularly informative. Regular soft drinks/cola accounted for 62.3% of estimated strict SSB intake by mass, while the remaining 37.7% originated from other categories, principally squash/cordials, sports drinks and energy drinks, with an additional contribution from sugar-sweetened fruit drinks. These findings illustrate the importance of including multiple beverage categories when characterising source-specific SSB exposure.

This also has implications for epidemiological classification. Broad categories such as “soft/energy/sports drinks” may combine sugar-containing products with diet, zero-sugar or otherwise non-caloric variants. Such grouping creates exposure heterogeneity and may attenuate associations with biological outcomes. The source-resolved approach used here does not eliminate dietary measurement error, but it reduces one specific and avoidable component of classification error: treating nutritionally dissimilar beverages as if they represented the same exposure. Sweet taste should likewise not be used as a proxy for free-sugar exposure. Low- and non-caloric sweeteners can maintain sweetness without corresponding sugar intake, and recent UK evidence indicates that liking for sweet taste does not map straightforwardly onto free-living sugar intake [35]. This further supports the exclusion of diet/no-sugar beverages from the strict SSB definition rather than treating broad soft-drink categories as proxies for sugar exposure.

The separate analysis of 100% fruit/vegetable juice provides a useful demonstration of this principle. Juice contributed to free-sugar exposure but was not classified as a strict SSB. In the present study, 83.8% of participants consumed 100% fruit/vegetable juice, and none of the three secondary adjusted associations with BMI, body-fat percentage or visceral-fat index reached statistical significance. These results are compatible with the complexity of the wider evidence. A 2024 systematic review and meta-analysis including 42 eligible studies found that 100% fruit juice was associated with small BMI increases in children, whereas evidence in adults was substantially more equivocal: associations in prospective cohorts varied according to energy adjustment, and pooled adult randomised trials did not demonstrate a significant effect on body weight [36].

The juice findings are therefore useful not because they establish benefit or harm in this cohort, but because they show why free-sugar classification and beverage taxonomy should not be collapsed into a single category. An exposure may qualify as a source of free sugars while having different nutrient composition, dietary context and epidemiological evidence from an SSB containing added sugars.

### 4.3. Interpreting the Adiposity Findings in the Context of the Wider Evidence

The adjusted associations observed in this cross-sectional cohort should be interpreted alongside longitudinal and experimental evidence on dietary sugars and adiposity. Randomised and prospective evidence synthesised by Te Morenga et al. demonstrated that increases and reductions in dietary sugars under *ad libitum* conditions are accompanied by corresponding changes in body weight [11]. Systematic reviews of SSB consumption have likewise associated higher intake with subsequent weight gain and cardiometabolic risk [12,13,14]. More recent evidence indicates that replacing SSBs with non-caloric alternatives can result in small favourable effects on weight [15].

The present findings do not challenge that literature because the underlying causal question differs fundamentally. A cross-sectional analysis estimates whether individuals with differing current exposures also differ in contemporaneous adiposity after adjustment for specified covariates. It does not estimate the effect of sustained exposure over time, nor does it estimate the consequence of experimentally changing SSB or free-sugar consumption.

Adipose-tissue accumulation reflects energy balance integrated over prolonged periods. A current estimate of habitual diet may only imperfectly represent the exposure history responsible for present body composition. This is especially relevant to a population aged only 18–22 years, in whom adult dietary behaviours may have been established relatively recently. Exposure and phenotype need not appear simultaneously: substantial SSB consumption at age 20 does not necessarily imply that sufficient time has elapsed for modest chronic energy imbalances to generate detectable differences in body fat or visceral adiposity.

The present models estimate cross-sectional associations conditional on the specified covariates, rather than causal effects. Although none of the primary associations reached statistical significance, the confidence intervals remained compatible with effects in either direction, particularly for body-fat percentage. These findings should therefore be interpreted in terms of the estimated effects and their precision rather than statistical significance alone.

Reverse causation may further weaken cross-sectional associations. Students with greater adiposity or greater health awareness may already have reduced their consumption of sugar-containing beverages, switched to low/no-sugar products or changed other aspects of their diet. Such behaviour could attenuate a positive exposure–adiposity association even if higher cumulative SSB intake had previously contributed to weight gain.

Age-related restriction of phenotype is particularly relevant to the visceral-fat analysis. Previous adult research has associated SSB consumption with visceral adiposity [16], while Ma et al. reported greater accumulation of visceral adipose tissue during six years of follow-up among participants consuming more SSBs [17]. These investigations address a different exposure duration and generally older populations.

By contrast, visceral-fat index in the present cohort ranged only from 1 to 9, entirely below the manufacturer-defined elevated range. This represents substantial range restriction in the outcome. When nearly all participants occupy a relatively favourable and compressed portion of a physiological scale, the capacity to identify between-person exposure gradients is necessarily reduced. The present visceral-fat findings are therefore compatible with, rather than contradictory to, evidence showing gradual accumulation of visceral adipose tissue over years.

Energy adjustment provides another important interpretative issue. Total energy was included in the primary models because the intended estimand was the association of dietary composition or beverage exposure with adiposity beyond overall reported intake. However, an important mechanism through which SSBs may promote weight gain is precisely through additional energy intake and incomplete caloric compensation. SACN concluded that energy delivered as sugars in beverages may be incompletely compensated for by reductions elsewhere in the diet [8]. Adjustment for energy could therefore statistically remove part of a pathway through which SSB exposure acts.

Sensitivity models without total energy adjustment yielded estimates for strict SSB and free-sugar exposures that were broadly similar in direction and magnitude to those from the primary models (Supplementary Table S7). The consistency of these estimates indicates that the observed associations were not highly sensitive to the inclusion of total energy in the models.

The recent UK randomised study by Boxall et al. provides complementary rather than contradictory evidence [37]. Dietary recommendations designed to reduce free-sugar intake produced meaningful reductions in exposure and modest anthropometric differences over 12 weeks. An intervention trial asks whether actively modifying exposure changes subsequent outcomes; the present study asks whether habitual exposure at one time point differentiates contemporaneous phenotype. The estimands, temporal ordering and susceptibility to confounding are therefore fundamentally different. Evidence from intervention studies therefore provides complementary information on the effects of reducing free-sugar intake, whereas the present cross-sectional analysis does not establish the effectiveness of such interventions.

The absence of cross-sectional associations is also not inconsistent with the biological rationale underlying current guidance. SACN’s recommendations are informed not only by body weight but by evidence concerning total energy intake, dental caries and metabolic outcomes, including type 2 diabetes risk associated with SSB consumption [8]. The public-health significance of a high free-sugar exposure cannot therefore be judged solely by whether BMI is elevated in a sample of 20-year-old students.

The robustness analyses further demonstrated that the estimated associations were broadly consistent across alternative exposure definitions and model specifications. Results were unchanged when free sugars were expressed in g/day; when SSB intake was analysed on its original g/day scale; when SSB exposure was represented as consumer versus non-consumer; when total energy adjustment was removed; when the complete *N* = 111 cohort was retained; after influence-based exclusions; and when the three ambiguous beverages were conservatively incorporated into the SSB exposure. Quadratic models also provided no evidence that a simple departure from linearity explained the primary results.

### 4.4. Historical Policy Context and Evidence from University Food Environments

The timing of the present study provides a relevant historical context for interpreting its findings. Dietary data were collected in early 2016, before implementation of the UK Soft Drinks Industry Levy (SDIL) in April 2018. The study therefore provides a source-resolved characterisation of beverage intake preceding substantial reformulation of the UK soft-drink market. This distinction is important when interpreting historical beverage classifications alongside subsequent developments in sugar-reduction policy.

Evidence indicates that the SDIL stimulated extensive reformulation. Controlled interrupted time-series analysis showed a marked reduction in the proportion of potentially levy-eligible beverages containing >5 g sugar/100 mL following announcement and implementation of the levy [38]. Subsequent government monitoring reported a 46% reduction in sales-weighted mean sugar content in drinks within the scope of the levy between 2015 and 2020 [39].

Importantly, evidence of change extends beyond product composition. Analysis of individual-level NDNS data estimated that, relative to the counterfactual trend, whole-diet free-sugar intake 11 months after SDIL implementation was lower by approximately 4.8 g/day in children and 10.9 g/day in adults [40]. These findings cannot establish that every aspect of the change was attributable exclusively to the levy, but they demonstrate that structural beverage policy can coincide with nutritionally meaningful population-level changes.

This context has a direct bearing on our historical FoodName classification. A beverage purchased today may not contain the same amount of sugar as a product carrying the same commercial name during 2015–2016. Retrospective assignment based on present formulations would therefore risk systematic error precisely because reformulation has been one of the major consequences of UK sugar-reduction policy. Our conservative treatment of historical ambiguity is consequently not merely an analytical preference; it is necessary to preserve temporal validity.

The continued strengthening of the SDIL framework further demonstrates that beverage reformulation remains a contemporary policy priority. Current UK policy will lower the levy threshold and extend its scope from January 2028 [41]. The taxation framework and the epidemiological strict SSB definition used here are not identical constructs; nevertheless, both reflect a continuing shift towards altering the composition and availability of high-sugar beverages at the population level.

University food environments have been investigated as potential settings for dietary interventions, including approaches targeting food availability, pricing and purchasing behaviour. A systematic review by Li et al. concluded that taste, availability and price were prominent influences on university students’ dietary choices, while living arrangements and socioeconomic circumstances also affected diet quality [42]. These findings identify potentially relevant environmental determinants of dietary behaviour that warrant further investigation in future university-based intervention studies.

More importantly, there is now interventional evidence that university food environments are modifiable. Barrett et al. systematically reviewed 55 studies describing 71 retail food-environment interventions in postsecondary settings [43]. Approximately two-thirds of interventions produced significant improvements in dietary behaviour. Interventions targeting product availability or convenience were particularly promising, with nine of 10 reporting improvements, while pooled evidence from interventions reporting purchased or consumed energy showed a reduction of approximately 7.9%. These findings provide empirical evidence supporting further evaluation of food-environment interventions in university settings.

Complementary evidence on individual determinants of SSB consumption comes from the systematic review by Calabro et al. [44], which examined socio-cognitive determinants of SSB consumption among young people and found that habit, behavioural intention and attitudes were particularly strongly related to the amount consumed, while intention and social norms were important for consumption frequency [44]. These findings identify behavioural determinants that could be investigated as potential targets for future interventions aimed at reducing sugar-sweetened beverage consumption among young adults.

Together, these external studies identify several potentially relevant approaches for future investigation, ranging from changes in beverage availability and purchasing environments to interventions addressing habitual consumption. Their effectiveness in the present university cohort was not evaluated, and they should therefore be considered potential avenues for future research rather than direct intervention recommendations arising from the historical findings.

### 4.5. Strengths, Limitations and Priorities for Future Research

Several characteristics strengthen the present study. First, the narrow age range reduces biological heterogeneity and focuses specifically on emerging adulthood. Second, the cohort was geographically diverse. Third, height and weight were measured rather than self-reported, and adiposity assessment extended beyond BMI to include BIA-derived body-fat percentage and visceral-fat index.

Fourth, the dietary exposure was reconstructed directly from the original records rather than inferred retrospectively from summary statistics. The native free-sugar field was preserved, all 441 FoodNames were audited, diet/no-sugar products were separated from strict SSBs, 100% juice was retained as a distinct secondary exposure, ambiguous beverages were explicitly documented, and source-level sensitivity analyses were conducted.

Fifth, the statistical framework was deliberately conservative. The primary outcomes and exposures were specified, HC3 robust standard errors were used to account for potential heteroskedasticity, and multiplicity was addressed across the defined six-test primary family. Alternative parameterisations, energy-adjustment sensitivity analyses, influence diagnostics and exposure-classification checks supported the stability of the estimated associations across model specifications.

Sixth, the analytical framework, including the primary dietary exposures, adiposity outcomes, adjustment covariates, and model specifications, was established before the statistical analyses were conducted. The use of a reproducible analytical workflow, supported by retained R scripts and definitive outputs, ensured consistency, transparency, and traceability of the reported results, tables, and figures.

These strengths should be interpreted alongside several constraints. The sample comprised 111 volunteers from a single English university and was predominantly female; it was not designed to provide nationally representative prevalence estimates, and the modest sample limits precision for small effects and subgroup interactions. The cross-sectional design also precludes temporal or causal inference, does not capture cumulative exposure and remains susceptible to reverse causation. Furthermore, incomplete historical recruitment and screening records prevented reconstruction of the full participant flow and calculation of a formal response rate, limiting the assessment of potential selection bias.

Dietary exposure was self-reported using an adapted FFQ that was not independently validated specifically for free-sugar or source-resolved SSB assessment in this cohort. FFQs can characterise reported dietary intake over the specified reference period, although recall, portion-size estimation and food-composition assignment may introduce measurement error [45], potentially attenuating diet–adiposity associations. BIA likewise provides estimates rather than direct anatomical measurements and is not interchangeable with DXA or MRI [21,22]; the restricted visceral-fat range of 1–9 further reduced sensitivity to detect small between-participant differences.

The historical nature of the data also requires careful interpretation. Beverage formulation, availability, marketing and policy have changed substantially since 2015–2016, particularly following the SDIL; the absolute SSB values reported here should therefore not be interpreted as contemporary 2026 prevalence estimates. Conversely, this timing provides a distinctive pre-implementation snapshot of the UK beverage environment and strengthens the rationale for avoiding retrospective product classification based on current formulations.

Residual confounding remains possible because physical activity and relevant socioeconomic and behavioural factors were not included in the primary adjustment set. Furthermore, free-sugar, total energy and beverage-intake estimates originated from the same FFQ, so correlated reporting and portion-size errors could affect percentage-energy estimates and the resulting associations. The observed dispersion in reported energy intake and the upper range of free-sugar exposure should be considered in this context.

Future research should build on the exposure specificity of the present work rather than merely reproduce the same cross-sectional design in a larger sample. Multicentre prospective cohorts should recruit students at or before entry to university and repeatedly assess source-specific beverage consumption and free-sugar intake using validated dietary-assessment methods, alongside repeated body-composition measurements throughout higher education.

Such studies should include objective or repeated measures of physical activity; socioeconomic position; housing and cooking facilities; food security; smoking and alcohol intake; and, where relevant, migration and acculturation variables. This would allow substantially more complete confounder control and enable investigation of whether determinants of beverage exposure differ among student subpopulations without using geographical origin as a proxy for unmeasured social or cultural characteristics.

Repeated assessment would also make it possible to examine within-person change, which is arguably the central unanswered question emerging from the present study. Rather than asking whether students with higher SSB consumption have higher BMI at age 20, future research should determine whether sustained or increasing SSB/free-sugar exposure between ages 18 and 22 predicts subsequent change in body weight, body-fat percentage or visceral adiposity.

Direct body-composition methods such as DXA or MRI would be particularly valuable for examining regional adipose-tissue accumulation. The longitudinal evidence relating SSB intake to visceral adiposity suggests that such outcomes may be more informative when followed over several years than when evaluated cross-sectionally within a metabolically young population [16,17].

Repeated *FoodName*-level or product-level dietary data would additionally enable direct evaluation of reformulation. This is particularly relevant in the UK, where the same beverage category—and potentially the same commercial product name—may now represent a substantially different sugar exposure from that recorded before the SDIL. Linking product composition to longitudinal dietary records could therefore help distinguish behavioural substitution from manufacturer-driven reformulation.

## 5. Conclusions

This historical cross-sectional study characterised free-sugar and source-resolved sugar-sweetened beverage (SSB) intake among 111 young adults aged 18–22 years attending an English university in early 2016. Median free-sugar intake represented 8.20% of total reported energy, 33.3% of participants had intakes ≥ 10%, and 81.1% consumed strict SSBs. Regular soft drinks/cola accounted for 62.3% of estimated strict SSB intake by mass, while other beverage categories collectively contributed 37.7%, highlighting the value of distinguishing individual beverage sources.

In the primary adjusted analyses (*n* = 109), none of the six prespecified associations between free sugars or strict SSB intake and BMI, BIA-derived body-fat percentage or visceral-fat index reached statistical significance. However, the confidence intervals encompassed potentially meaningful associations in either direction. For example, a 1-SD higher log1p-transformed strict SSB intake was associated with a +0.87-percentage-point difference in body fat (95% CI −1.14 to 2.88). Sensitivity analyses yielded broadly consistent estimates across alternative exposure definitions and model specifications. The findings should therefore not be interpreted as demonstrating the absence of an association.

By integrating nutrient-level free-sugar assessment with an auditable FoodName-level classification of beverage sources and measured body-composition outcomes, this study provides a reproducible characterisation of dietary sugar exposure before implementation of the UK Soft Drinks Industry Levy. These historical estimates do not represent current prevalence or establish the effects of dietary interventions. Larger prospective studies across university populations, incorporating validated source-specific dietary assessment and repeated body-composition measurements, are warranted to investigate the longer-term relationships between free-sugar and SSB exposure and adiposity.

## Figures and Tables

**Table 1:** The characteristics of the study population (*N* = 111) and dietary sugar/beverage exposures.

Characteristic	Level	Summary
Age		20.4 ± 1.2; median 20.0 (20.0–21.0)
Sex	Female	78 (70.3%)
	Male	33 (29.7%)
Geographical background	Africa	41 (36.9%)
	Asia	41 (36.9%)
	Europe	27 (24.3%)
	Other	2 (1.8%)
Total energy intake, kcal/day		2524.1 ± 1256.1; median 2245.4 (1665.8–3108.5)
Total sugars, g/day		144.0 ± 91.7; median 121.7 (83.4–160.4)
Free sugars, g/day		64.2 ± 59.5; median 43.2 (27.9–80.1)
Free sugars, % total energy		9.81 ± 6.65; median 8.20 (5.38–12.28)
Strict SSB intake, g/day		343.2 ± 452.9; median 159.1 (45.4–483.9)
100% fruit/vegetable juice intake, g/day		43.2 ± 72.5; median 22.9 (1.4–46.4)
BMI, kg/m^2^		23.69 ± 4.09; median 23.35 (21.26–25.94)
Body fat, %		25.7 ± 10.4; median 24.8 (18.8–31.6)
Visceral-fat index		2.67 ± 2.02; median 2.00 (1.00–4.00)

Data are mean ± SD; median (IQR) for continuous variables and n (%) for categorical variables. Strict SSB = strict sugar-sweetened beverage exposure derived at FoodName level. The displayed geographical-background percentages sum to 99.9% because of rounding to one decimal place. The two participants in the “Other” geographical-background category are included in descriptive summaries.

**Table 2:** Source-resolved strict sugar-sweetened beverage consumption.

Beverage Source	Included in Strict SSB	Consumers, *n* (%)	Mean ± SD, g/Day	Median (IQR), g/Day	Contribution to Strict SSB, %
Strict SSB—total	Total	90 (81.1%)	343.2 ± 452.9	159.1 (45.4–483.9)	100.0
Regular soft drinks/cola	Yes	80 (72.1%)	213.9 ± 340.4	94.3 (0.0–262.0)	62.3
Squash/cordials	Yes	53 (47.7%)	66.7 ± 211.0	0.0 (0.0–45.1)	19.4
Sports drinks	Yes	17 (15.3%)	31.9 ± 95.4	0.0 (0.0–0.0)	9.3
Energy drinks	Yes	28 (25.2%)	19.3 ± 48.1	0.0 (0.0–17.9)	5.6
Sugar-sweetened fruit drinks	Yes	11 (9.9%)	11.4 ± 91.3	0.0 (0.0–0.0)	3.3
100% fruit/vegetable juice intake	No	93 (83.8%)	43.2 ± 72.5	22.9 (1.4–46.4)	

Strict SSB included regular soft drinks/cola, sugar-containing squash/cordials, sports drinks, energy drinks and sugar-sweetened fruit drinks. Consumer percentages and intake summaries were calculated for the full cohort (*N* = 111), including zero intakes among non-consumers. Beverage-source categories were not mutually exclusive. Contributions were calculated from cohort-level estimated SSB intake by mass and sum to 99.9% because of rounding. Consumer-only summaries are provided in Supplementary Table S4. Diet/no-sugar beverages and 100% fruit/vegetable juice were excluded from strict SSB intake.

**Table 3:** Multivariable-adjusted associations of free sugars and strict SSB intake with adiposity and body-composition outcomes.

Exposure	Outcome	Exposure Scale	*N*	B (95% CI)	Standardised β (95% CI)	*p*	BH-FDR *q*
Free sugars (% total energy)	BMI	per +5 percentage points	109	−0.065 (−0.509 to 0.379)	−0.021 (−0.164 to 0.122)	0.771	0.942
	Body fat (%)	per +5 percentage points	109	0.498 (−0.684 to 1.681)	0.063 (−0.086 to 0.212)	0.405	0.942
	Visceral-fat index	per +5 percentage points	109	−0.032 (−0.297 to 0.232)	−0.021 (−0.196 to 0.154)	0.810	0.942
Strict SSB	BMI	per +1 SD log1p(g/day)	109	0.037 (−0.963 to 1.037)	0.009 (−0.234 to 0.251)	0.942	0.942
	Body fat (%)	per +1 SD log1p(g/day)	109	0.872 (−1.138 to 2.881)	0.083 (−0.108 to 0.274)	0.392	0.942
	Visceral-fat index	per +1 SD log1p(g/day)	109	−0.100 (−0.576 to 0.376)	−0.050 (−0.287 to 0.187)	0.677	0.942

Primary models used HC3 robust standard errors and were adjusted for age, sex, geographical background, and log-transformed total energy intake. Formal inference included Africa, Asia, and Europe (*n* = 109); the two participants classified as Other were excluded from the primary adjusted models. Free sugars were scaled per 5-percentage-point increase in total energy. Strict SSB was modelled per 1-SD increase in log1p(g/day). Benjamini–Hochberg false-discovery-rate correction was applied jointly across the six prespecified primary tests.

## Data Availability

Derived non-identifiable analytical outputs, the FoodName-level beverage-classification audit, figure-source data, Supplementary Materials and reproducible R code supporting this study are available in Figshare at https://doi.org/10.6084/m9.figshare.33696679. The complete participant-level dataset is not publicly available because it contains human-participant information subject to ethical, institutional and data-protection requirements. Requests for additional de-identified information may be considered by the corresponding author in accordance with applicable ethical and institutional conditions.

## Supplementary Materials

The following supporting information can be downloaded at https://www.mdpi.com/article/10.3390/nu18213552/s1, Supplementary File S1 (XLSX): Table S1, FoodName-level beverage classification audit used to derive strict SSB and 100% fruit/vegetable juice exposures; Table S2, Extended descriptive statistics for dietary sugars and beverage exposures; Table S3, Descriptive dietary and adiposity measures by sex and geographical background; Table S4, Consumer-only intake summaries for source-resolved strict sugar-sweetened beverage subtypes; Table S5, Secondary multivariable-adjusted associations of 100% fruit/vegetable juice intake with adiposity and body-composition outcomes; Table S6, Prespecified sensitivity analyses for free sugars and strict SSB exposures; Table S7, Sensitivity analyses comparing primary exposure parameterisations with and without total-energy adjustment; Table S8, Robustness analysis including the three ambiguously classified beverage FoodNames in an expanded SSB exposure; Table S9, Primary-model diagnostics and additional robustness checks. Supplementary File S2 (DOCX): Figure S1, Distribution of the bioelectrical impedance analysis (BIA)-derived visceral-fat index in the study cohort (*N* = 111); Figure S2, Distribution of strict sugar-sweetened beverage (SSB) intake in 111 young adults attending an English university; Figure S3, Robustness of the associations between sugar exposures and adiposity/body-composition outcomes across the primary and principal sensitivity analyses.
