# Supplementary material for: Free Sugars and Source-Resolved Sugar-Sweetened Beverage Consumption in Geographically Diverse Young Adults at an English University (2015–2016): Associations with Adiposity and Body Composition
Source: Nutrients. 2026 Oct 28;18(21):3552. doi: 10.3390/nu18213552 (PMC13765343; doi:10.3390/nu18213552)
Supplement: Supplementary file 1 [file nutrients-18-03552-s001.zip › Supplementary File S2.pdf]

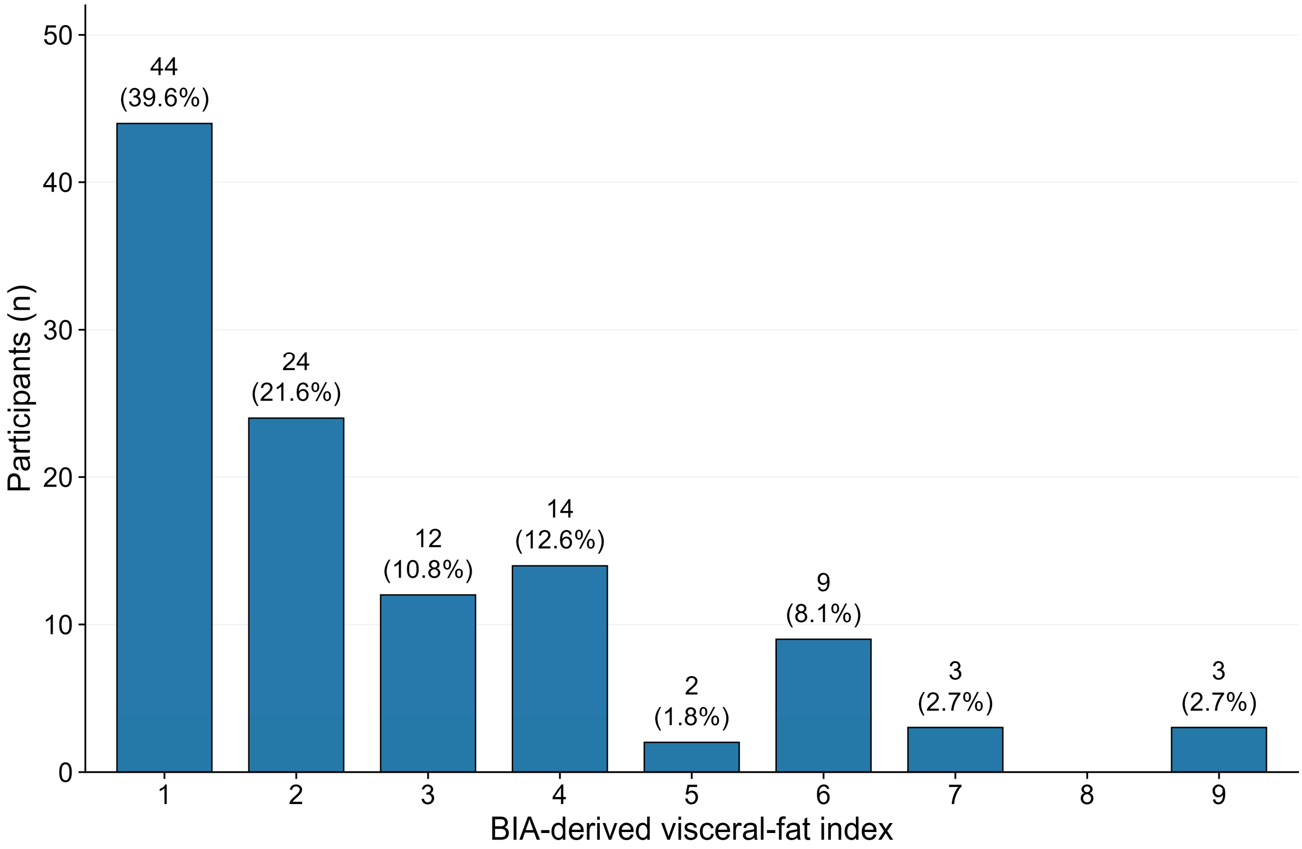

Figure S1. Distribution of the bioelectrical impedance analysis (BIA)-derived visceral-fat index in the study cohort (*N* = 111). Bars represent the number of participants at each index value, with percentages calculated relative to the full cohort. Values ranged from 1 to 9 (median 2; IQR 1–4), and 68 participants (61.3%) had an index of 1 or 2. No participant reached the manufacturer's elevated threshold (≥13).

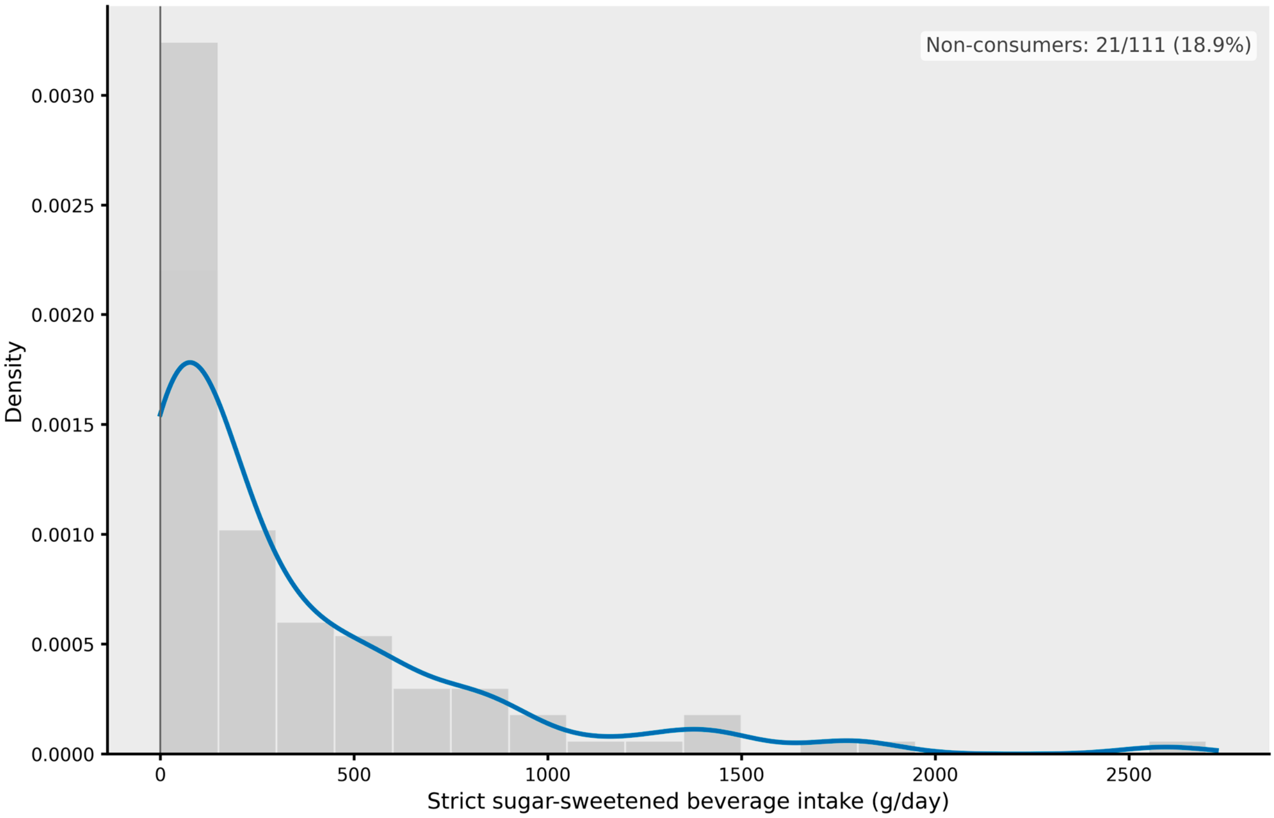

Figure S2. Distribution of strict sugar-sweetened beverage (SSB) intake in 111 young adults attending an English university. The histogram and kernel-density curve show strict SSB intake in g/day. Twenty-one participants (18.9%) were non-consumers (0 g/day). The pronounced right-skew and the presence of structural zeroes supported use of the log1p transformation, followed by standardisation, for strict SSB intake in the primary regression models.

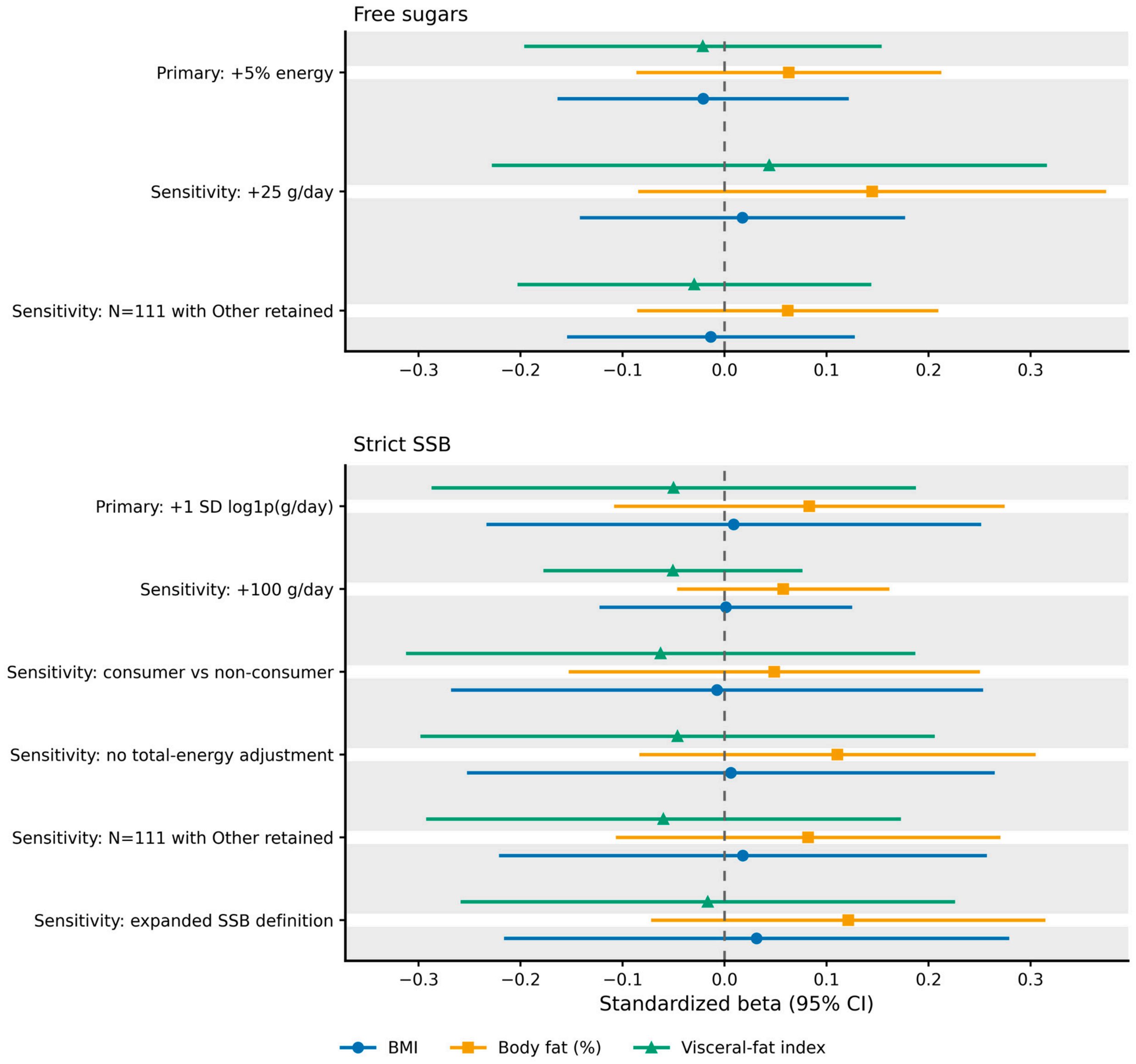

Figure S3. Robustness of the associations between sugar exposures and adiposity/body-composition outcomes across the primary and principal sensitivity analyses. Points represent standardised regression coefficients (β) and horizontal lines represent 95% confidence intervals for BMI, body fat (%) and visceral-fat index. The upper panel shows free sugars under the primary parameterisation (+5 percentage points of total energy), the alternative +25 g/day parameterisation and the analysis retaining the two participants in the Other geographical-background category (N = 111). The lower panel shows strict SSB intake under the primary parameterisation (+1 SD of log1p-transformed g/day), +100 g/day, consumer versus non-consumer, omission of total-energy adjustment, N = 111 with Other retained, and the expanded SSB definition including the three ambiguous beverage items. The vertical dashed line denotes the null. Because coefficients are standardised within each parameterisation, the figure is intended to show the direction, magnitude and stability of associations across model specifications rather than equivalence of raw-unit effects.
